# Detection of epileptic seizure based on entropy analysis of short-term EEG

**DOI:** 10.1371/journal.pone.0193691

**Published:** 2018-03-15

**Authors:** Peng Li, Chandan Karmakar, John Yearwood, Svetha Venkatesh, Marimuthu Palaniswami, Changchun Liu

**Affiliations:** 1 School of Control Science and Engineering, Shandong University, Jinan, Shandong, China; 2 School of Information Technology, Deakin University, Burwood, Victoria, Australia; 3 Department of Electrical & Electronic Engineering, University of Melbourne, Parkville, Victoria, Australia; 4 Centre for Pattern Recognition and Data Analytics (PRaDA), Deakin University, Geelong, Victoria, Australia; University of California San Diego, UNITED STATES

## Abstract

Entropy measures that assess signals’ complexity have drawn increasing attention recently in biomedical field, as they have shown the ability of capturing unique features that are intrinsic and physiologically meaningful. In this study, we applied entropy analysis to electroencephalogram (EEG) data to examine its performance in epilepsy detection based on short-term EEG, aiming at establishing a short-term analysis protocol with optimal seizure detection performance. Two classification problems were considered, i.e., 1) classifying interictal and ictal EEGs (epileptic group) from normal EEGs; and 2) classifying ictal from interictal EEGs. For each problem, we explored two protocols to analyze the entropy of EEG: i) using a single analytical window with different window lengths, and ii) using an average of multiple windows for each window length. Two entropy methods—fuzzy entropy (FuzzyEn) and distribution entropy (DistEn)–were used that have valid outputs for any given data lengths. We performed feature selection and trained classifiers based on a cross-validation process. The results show that performance of FuzzyEn and DistEn may complement each other and the best performance can be achieved by combining: 1) FuzzyEn of one 5-s window and the averaged DistEn of five 1-s windows for classifying normal from epileptic group (accuracy: 0.93, sensitivity: 0.91, specificity: 0.96); and 2) the averaged FuzzyEn of five 1-s windows and DistEn of one 5-s window for classifying ictal from interictal EEGs (accuracy: 0.91, sensitivity: 0.93, specificity: 0.90). Further studies are warranted to examine whether this proposed short-term analysis procedure can help track the epileptic activities in real time and provide prompt feedback for clinical practices.

## Introduction

Epilepsy affects approximately 9 million people in China [1] and more than 65 million people worldwide [2]. It is the fourth most common neurological disorder in the USA [2]. In Australia, the prevalence of epilepsy is between 0.6%-0.75% [3]. Nearly 80% of the people with epilepsy live in low- and middle-income countries, among which, however, over three fourths do not get the treatment they need [4]. To reduce this treatment gap, there is not only a need for well-trained healthcare providers, but related technologies/devices that can detect or even track epileptic activities reliably and cost-effectively are also required urgently.

Continuous electroencephalogram (EEG) monitoring allows uninterrupted assessment of brain activity [5] and thus makes the tracking of seizure events possible. In order for caregivers to take prompt action during monitoring, a rapid seizure onset or seizure attack detection is required which is, however, still challenging. It relies not only on accurate performance but also the compatibility of classification algorithms with inputs of short-term EEG data (e.g., length≤5 s). To the best of our knowledge, only very few, amongst the vast number of published studies, were initially designed for handling short-term data [6, 7]. The reason for using longer signal recordings may partly lie in the fact that the algorithms used in most previously published studies, e.g., empirical mode decomposition (EMD) [8], wavelet transform [9], and detrended fluctuation analysis (DFA) [10], require large numbers of data points for robust performance. Note that in [8], although the authors have claimed that their statistical features were extracted from the IMF (intrinsic mode function which is extracted using the EMD algorithm) segments of only 256 points (~1.5 s), their preprocessing (filtering signal using a Butterworth band-pass filter) and extraction of IMFs from EEG was done on the complete EEG recording.

In view of clinical practices, two important aspects of EEG based seizure detection can be described as: i) screening subjects with epilepsy from the normal cohort, i.e., classifying interictal and ictal EEG from normal EEG; and ii) detection of seizure in epileptic population, i.e., classifying ictal from interictal EEG. Based on one of the most widely used open access EEG data sets—the Bonn database [11, 12], the two problems can be specified as classifying: i) N (EEG during interictal phase recorded from the opposite hemisphere of the epileptogenic zone), F (EEG during interictal phase recorded from the epileptogenic zone) and S (EEG at ictal phase) from Z (normal EEG recorded with eyes closed), O (normal EEG recorded with eyes open); and ii) N, F from S. However, to the best of our knowledge, no publication has yet reported the results exactly in this way (published studies including [9, 10, 13–26] and for a review see [27]). Though various models that combined several classification problems have been developed, most of them were found to have only covered one of the two problems. Specifically, a few studies which targeted detection of epileptic seizure always used set S as one class and either one or a combination (e.g., ZNF, ONF, or very occasionally ZONF) set from sets Z, O, N, and F [9, 13–16] (a detailed list regarding which one or what combination was used could be found in [9]). Some studies have worked on differentiating EEG at the ictal phase from those at the interictal phase. However, only one set from the interictal class (either N or F) along with set S were used to develop the models [10, 17, 18]. There are also a couple of studies which considered three-class (normal, interictal, and ictal) models [19–26], all of which used one set from the two EEG sets in both normal and interictal groups except [8, 23] which applied all five data sets. Though these three-class models may potentially satisfy the conventional clinical requirements, when taking continuous monitoring into account, e.g., for the online tracking and prompting of epileptic activities, their applicability may need to be further validated since all of them were developed based on long-term recordings with 4,097 sampling points (23.6 s).

Regarding features used to characterize EEG, nonlinear properties have attracted increasing attention nowadays since nonlinearity is believed to be inherent in physiological processes [28]. Various entropy measures, i.e., approximate entropy (ApEn) [29], sample entropy (SampEn) [30], permutation entropy [31], symbolic dynamics based entropy [32], and fuzzy entropy (FuzzyEn) [17], have been favored since they were capable of providing estimations of complexity, a nonlinear dynamical biomarker for healthy physiology [33], based on data of limited length. Recently, we established a new entropy method based on the distribution of inter-vector distances in order to achieve high robustness for extremely short data recordings [34]. This distribution entropy (DistEn), which acts as a new member of the entire family of entropy measures, has shown extraordinarily good performance compared with traditional algorithms in several fields [34–37].

Most recently, we have applied DistEn and SampEn on the Bonn database to analyze 5-s EEG and found that DistEn worked well for classifying interictal EEG from normal, ictal EEG from normal, and ictal from interictal EEG, whereas SampEn failed in one of the three classification problems (ictal from interictal EEG) [6]. Additionally, we also reported that DistEn could still work when using 1-s EEG segment with a protocol of moving analytical windows [7]. Based on those previous findings, we aim to develop an entropy-based short-term EEG classification model, which is suitable for clinical settings and can be used to prompt the diagnosis of epileptic conditions or interventions during seizure for epileptic patients. We have decided to use entropy based approaches since they are applicable for probing the dynamics with data of limited length [34]. The short-term analyses we explored here will offer model compatibility for clinical settings as well as provide capacity for early intervention during seizure attack.

In this study, we will use FuzzyEn and DistEn methods since both of them are defined for any given data length (even as short as 1 s though FuzzyEn may vary severely) in contrast to SampEn. Besides, although ApEn is also defined for short data length, it was left out due to the bias of the measure (especially for short-term data). We have decided not to use permutation entropy or symbolic dynamics based entropy since to the best of our knowledge, no systematic study has been done yet regarding their applicability or application to short or extremely short physiological time-series.

To develop the model with the above-mentioned capacity, the challenge is to determine the optimal minimum EEG length under which a reasonably high accuracy could be achieved. We will apply two protocols to do this: i) entropy of a single window with length varying from 1 to 23 s (almost the complete recording length of the used database); and ii) average entropy of multiple windows with length *l* varying from 1 s to a certain length *x* s based on the results of protocol i) with overlapping of (*l* − 1) s. For both protocols, the performance will be measured as the capacity for distinguishing: i) normal from ictal and interictal EEGs (represented below by “epileptic group” for short); and ii) ictal from interictal EEGs.

## Methods

### Description of EEG data

The EEG data used in this study came from the Bonn database [12] which is publicly accessible online [11]. It is comprised of 500 single-channel EEG recordings sampled at 173.61 Hz with duration of 23.6 s each. They are categorized into five groups (classes Z, O, N, F, and S) and each group consists of 100 recordings. Classes Z and O are surface EEG data collected from five healthy volunteers using the standardized 10–20 electrode placement scheme in awake and relaxed state with their eyes open and closed, respectively. Classes N, F, and S were collected from five epileptic patients using intracranial electrodes. Signals in N and F were recorded from the opposite hemisphere and the hemisphere of the epileptogenic zone, respectively, during only seizure-free (interictal) periods. Signals in S were collected during the seizure attacks (ictal period). Prior to the following data analyses, all raw EEG recordings were filtered by a 20-order finite impulse response (FIR) band pass filter with cut-off frequencies of 0.53 and 40 Hz [12].

### Analysis protocols

**Fig 1:**
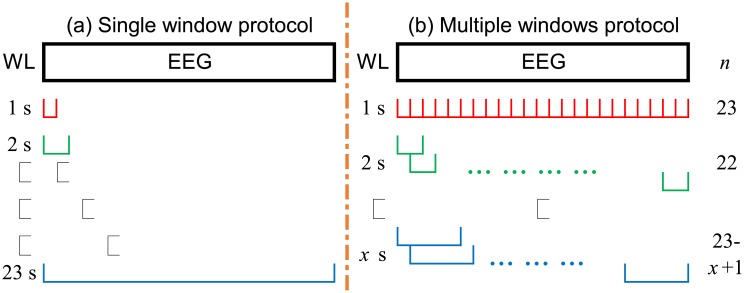
Analysis protocols. WL: window length. *n*: number of windows.

Two protocols were proposed for analysis in this study as shown schematically in Fig 1.

#### Single window protocol (SP)

Entropy (either FuzzyEn or DistEn) was calculated using short segments with length varying from 1 to 23 s. Segments always started from the very beginning of each recording, as indicated in Fig 1(a).

#### Multiple windows protocol (MP)

Entropy was obtained by averaging the entropy results over multiple segments. The length of segments varied from 1 s to a certain length *x* s based on results of SP. For each specific length *l*, the window shifted from the very beginning of the signal recording until (23 − *l*) s with an overlap of (*l* − 1) s between subsequent windows, as described in Fig 1(b). Note that in this protocol, the numbers of moving windows were different for different segment lengths.

### Algorithms of FuzzyEn and DistEn

#### Fuzzy entropy (FuzzyEn)

FuzzyEn is a refined algorithm for SampEn based on fuzzy logic. By definition, it does not count on the absolute probability of similar vectors according to the hard thresholding criterion as applied in SampEn. Instead, FuzzyEn estimates the probability that two vectors are similar based on the fuzzy membership function. Briefly, the FuzzyEn algorithm for a time-series of *N* points {*u*(*i*), 1 ≤ *i* ≤ *N*} can be summarized as follows:

1. State space reconstruction: Form (*N* − *mτ*) vectors **X**(*i*) by **X**(*i*) = {*u*(*i*), *u*(*i* + *τ*), ⋯, *u*(*i* + (*m* − 1)*τ*)}, 1 ≤ *i* ≤ *N* − *mτ*. Here *m* indicates the embedding dimension and *τ* the time delay.
2. Ranking similar vectors: Define the distance between **X**(*i*) and **X**(*j*) (1 ≤ *i*, *j* ≤ *N* − *mτ*, *i* ≠ *j*) by *d*_*i*,*j*_ = max(|*u*(*i* + *k*) − *u*(*j* + *k*)|, 0 ≤ *k* ≤ *m* − 1). Calculate the average probability that vectors **X**(*j*), *j* = 1, 2, ⋯, *N* − *mτ* and *j* ≠ *i* are similar to **X**(*i*) in terms of degree of membership using:
  $$A_{i}^{\left(m\right)}\left(r\right)=\frac{1}{N-m\tau }\Sigma_{j=1,j\neq i}^{N-m\tau }e^{-\text{ln}\left(2\right){\left(\frac{d_{i,j}}{r}\right)}^{2}}.$$ (1)
  Similarly, we define $A_{i}^{\left(m+1\right)}(r)$ as the counterpart when the subsequent point was included in the vectors. Here *r* indicates the threshold parameter.
3. Calculation: The FuzzyEn value of the time-series {*u*(*i*)} can be calculated by
  $$\text{FuzzyEn}\left(m,\tau ,r\right)=-\text{ln}\frac{\sum_{i=1}^{N-m\tau }A_{i}^{\left(m+1\right)}\left(r\right)}{\sum_{i=1}^{N-m\tau }A_{i}^{\left(m\right)}\left(r\right)}.$$ (2)

#### Distribution entropy (DistEn)

DistEn was initially proposed to alleviate the parameter-dependence and unrobustness of ApEn and SampEn especially when being applied to small data sets. It takes full advantage of the state space counterpart of the under-analyzed time-series by quantifying the distribution characteristics of the inter-vector distances. For the time-series {*u*(*i*), 1 ≤ *i* ≤ *N*}, DistEn can be estimated as follows:

1. State space reconstruction: Form (*N* − (*m* − 1)*τ*) vectors **X**(*i*) by **X**(*i*) = {*u*(*i*), *u*(*i* + *τ*), ⋯, *u*(*i* + (*m* − 1)*τ*)}, 1 ≤ *i* ≤ *N* − (*m* − 1)*τ*. Here *m* indicates the embedding dimension and *τ* the time delay.
2. Distance matrix construction: Compute the inter-vector distances (distances between all possible combinations of **X**(*i*) and **X**(*j*)) by *d*_*i*,*j*_ = max(|*u*(*i* + *k*) − *u*(*j* + *k*)|, 0 ≤ *k* ≤ *m* − 1) for all 1 ≤ *i*, *j* ≤ *N* − *m*. The distance matrix is denoted as **D** = {*d*_*i*,*j*_}.
3. Probability density estimation: Estimate the empirical probability density function of the distance matrix **D** by the histogram approach with a fixed bin number of *B*. The probability of each bin can be denoted as {*p*_*t*_, *t* = 1, 2, ⋯, *B*}. Note here elements with *i* = *j* in **D** are excluded in the estimation.
4. Calculation: The DistEn value of the time-series {*u*(*i*)} can be calculated by
  $$\text{DistEn}\left(m,\tau ,B\right)=-\frac{1}{{\text{log}}_{2}\left(B\right)}\Sigma_{t=1}^{B}p_{t}{\text{log}}_{2}\left(p_{t}\right).$$ (3)

#### Selection of input parameters

FuzzyEn is a function of *m*, *τ*, and *r*, whereas DistEn a function of *m*, *τ*, and *B*, as specified above. In this study, we were not exploring the effects of input parameters on either algorithm. Therefore, we chose to use those commonly recommended assignments for *r* and *B*, i.e., *r* = 0.15 * *sd* (*sd* indicates the standard deviation of the time-series under analysis) [38], and *B* = 64 [6, 34]. The embedding dimension *m* and time delay *τ* were determined jointly based on a differential entropy method [39]. Our analysis resulted in an optimal range of *m* ∈ [2,5] and *τ* ∈ [8,12], respectively. However, we did not apply all the possible combinations of *m* and *τ* in this study. Instead, *m* = 3 and *τ* = 3 were used because our recent study found that this combination works well for both algorithms (in that study we applied all the combinations, see [6] for details).

### Statistical analysis

Area under the receiver operating curve (AUC) was applied to test the ability of FuzzyEn and DistEn as measures for distinguishing: 1) normal from epileptic group (i.e., interictal and ictal EEG); and 2) ictal from interictal EEG. AUC can be a value from 0.5 to 1 and a smaller value indicates less discriminatory power.

**Fig 2:**
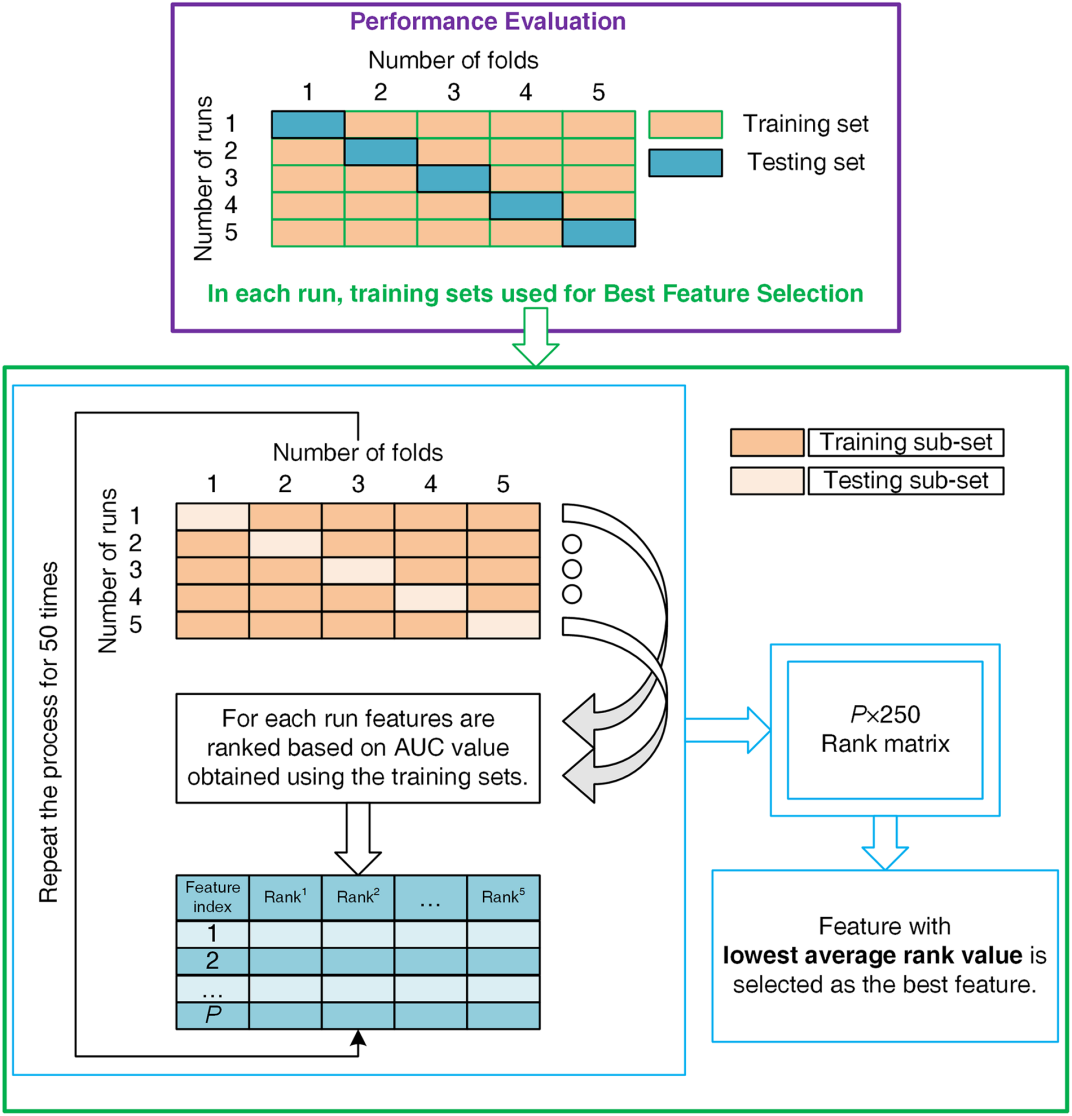
Performance evaluation and feature selection processes.

The quadratic discriminant (QD) classifier was applied to test the ability of FuzzyEn and DistEn features in detecting epileptic subjects from normal subjects at the first stage and then classifying ictal EEG from interictal at the second stage. A 5-fold cross-validation scheme was adopted to evaluate the generalization ability of the classifiers (Fig 2). Cross-validation procedures have been used in a number of classification evaluations, particularly for limited data sets [40]. In this scheme, the data set was uniformly divided into five subsets, maintaining the positive (epileptic or ictal) and negative (normal or interictal) class ratio. For the cross-validation, one set was used for testing and the remaining 4 subsets were used to train the classifiers. This was repeated for the remaining subsets so that all subsets were used as the testing sample. The feature selection method to select the single best feature from all FuzzyEn and DistEn features was embedded in the classification process, i.e., the single best feature was selected during each iteration of the cross-validation approach and the feature selection was performed using the training samples. A 5-fold partitioning scheme was used for dividing the training data uniformly into five subsets and at each run, four subsets of them were used for calculating the AUC value for each feature. Once all AUC values were calculated, features were ranked (1, 2, …, *P*) in descending order of AUC values, where *P* is the total number of features. Thus, the feature with the highest AUC value was given a rank value 1 and multiple features with same AUC values were given the same rank value. After one complete 5-fold partitioning scheme, there were five rank values for each feature and the feature rank matrix is of order *P* × 5. We have repeated the partitioning process 50 times to reduce the effect of randomization, which resulted in the final feature rank matrix of order *P* × 250. Finally, the rank of each feature was calculated by averaging all rank values of that feature and the feature with lowest average rank value was selected as the single best feature. After selecting the single best feature of each entropy feature group, they were used both independently and jointly to train the classification model and validate the developed model using test data. After all repetitions the classification results were obtained for the complete data set.

The following three measures, i.e., accuracy, sensitivity, and specificity, were used to assess the performance of the classifiers [41, 42]:

$$Accuracy=\frac{TP+TN}{TP+FP+TN+FN}\times 100,$$ (4)

$$Sensitivity=\frac{TP}{TP+FN}\times 100,$$ (5)

$$Specificity=\frac{TN}{TN+FP}\times 100,$$ (6)

where, *TP* is the number of true positives, i.e., the classifier identifies a patient that was labeled as epileptic (classification problem 1) or ictal (classification problem 2); *TN* is the number of true negatives, i.e., the classifier identifies a patient that was labeled as normal (problem 1) or interictal (problem 2); *FP* is the number of false epileptic or ictal identifications; and *FN* is the number of false normal or interictal identifications. Accuracy indicates overall detection capacity; sensitivity is defined as the ability of the classifier to accurately recognize epileptic or ictal, whereas specificity indicates the classifier’s ability not to generate a false negative (normal or interictal).

All statistical analyses were performed using MATLAB R2014b (The MathWorks Inc., Natick, Massachusetts, USA).

## Results

**Fig 3:**
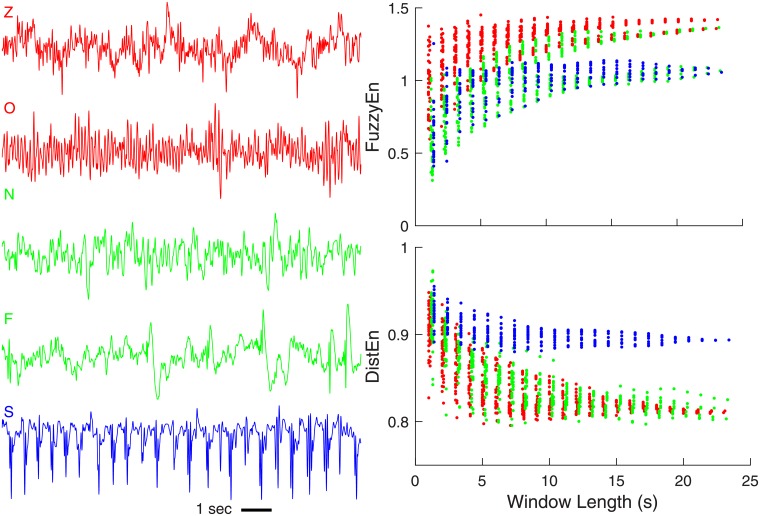
Exemplary EEG recordings from each of the five groups and their corresponding FuzzyEn and DistEn results calculated based on analysis protocol MP.

The left panels of Fig 3 show five exemplary EEG recordings with each coming from one of five groups, i.e., groups Z, O, N, F, and S. Their FuzzyEn and DistEn results calculated based on protocol MP are shown in the right two panels of Fig 3. Below we presented the analysis results based on the SP and MP protocols, as well as the classification results, separately in different sub-sections.

### Performance based on single window (protocol SP)

**Fig 4:**
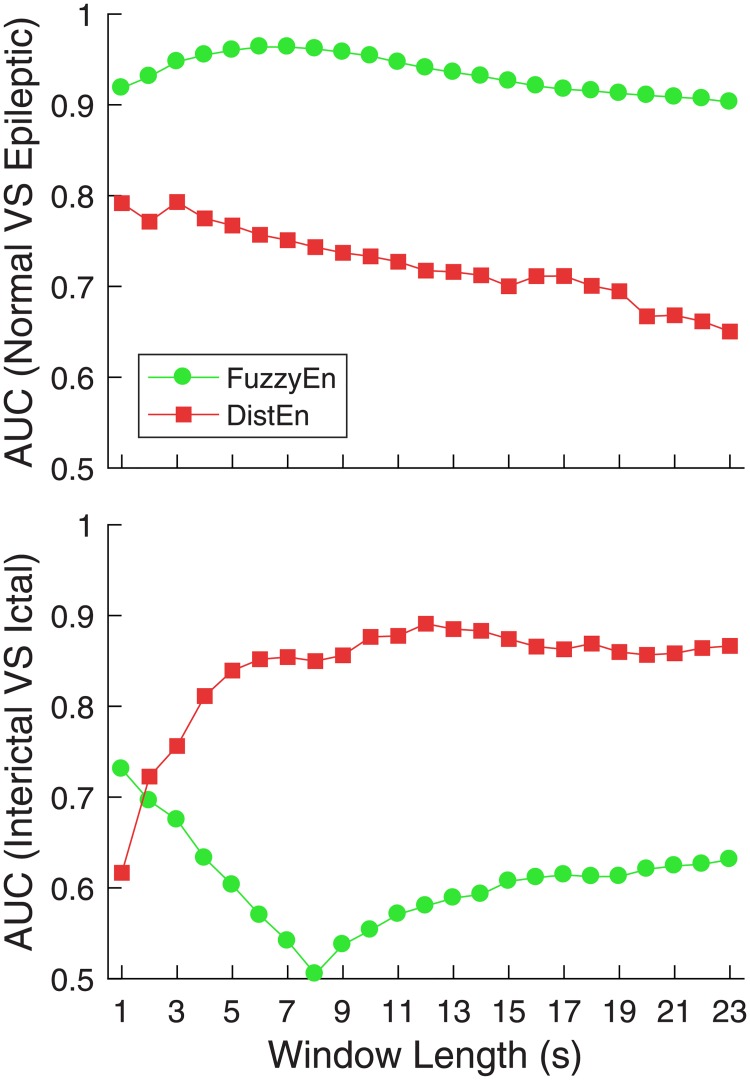
AUC results of analysis using single window protocol.

FuzzyEn showed good performance with AUC level consistently ≥ 0.9 for distinguishing normal subjects from the epileptic group; the performance was better with longer window length *n* initially (from *l* = 1 to 5 s) and was maintained at a high AUC level (~0.95). However, for distinguishing ictal from interictal EEG, FuzzyEn was not effective since the AUC levels were always lower than 0.7 no matter how long the window length was (Fig 4 and S1 Fig).

DistEn displayed totally different performance as compared with FuzzyEn. For distinguishing normal subjects from epileptic group, DistEn showed a relatively high AUC level (~0.8) initially (from *l* = 1 to 3 s) and then the AUC went down to < 0.7 slowly when the window length *n* was larger than ~15 s. However, it showed better performance for distinguishing ictal from interictal EEG with higher AUC levels (≥ 0.85), though initially with shorter window length (≤ 3 s) the AUC levels were lower than 0.8 (Fig 4 and S2 Fig).

For both algorithms, the best performance could be reached, or had already been reached, when the window length was 5 s. In addition, considering that we were targeting models based on short-term EEG, we applied window lengths ofup to *x* = 5 *s* in the MP protocol.

### Performance based on averages over multiple windows (protocol MP)

**Fig 5:**
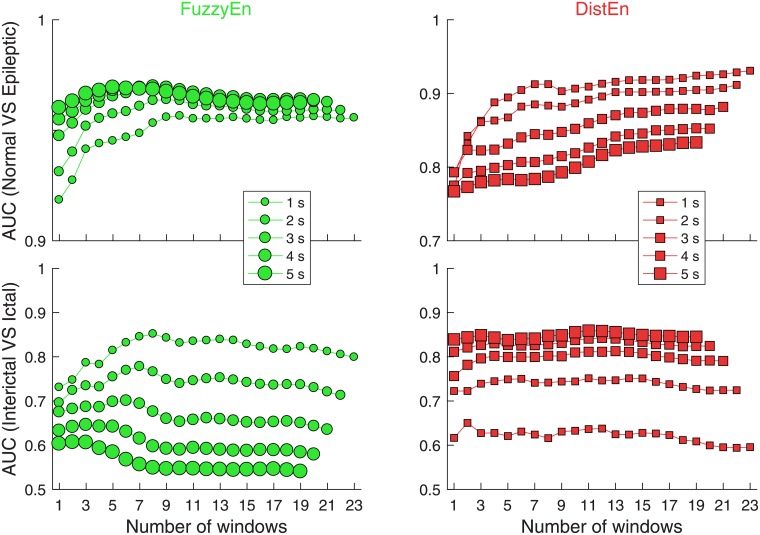
AUC results of analysis based on averaging over multiple windows.

Overall, as shown in the left two panels of Fig 5, for classifying normal from epileptic group, with the increase of window length, performance of FuzzyEn either became better (number of windows *n* ≤ 5) or remained (*n* > 5). For classifying ictal from interictal EEG, FuzzyEn showed worse performance with the increase of window length no matter how many windows were averaged. Specifically, for both classification tasks, when the window length was 1-s, the AUC values of FuzzyEn had an initial increase with the number of windows *n* and became saturated (without showing obvious increase) at *n* = ~5. For the two classification tasks using longer windows (i.e., 2–4 s), similarly the AUC results increased initially and then became unchanged or even reduced. The number of windows at which AUC became saturated shifted forward with the increase of window length.

The right two panels of Fig 5 show the AUC results of DistEn for the two classification tasks. The performance happened to be opposite to that of FuzzyEn. Specifically, for classifying normal from epileptic using a 1-s window, AUC values show an initial increase from *n* = 1 up to ~5 and became saturated without dramatic increase afterwards. For other window lengths from 2 to 5 s, the performance was similar except that the corresponding overall AUC values were reduced gradually. For classifying ictal from interictal EEG, the AUC values were almost unchanged for all *n* and the overall AUC values corresponding to different window lengths increased gradually from 1 to 5 s.

The detailed FuzzyEn and DistEn results did not follow a normal distribution and summarized in term of median±interquartile range (S3 and S4 Figs).

### Optimal feature selection and classification performance

**Fig 6:**
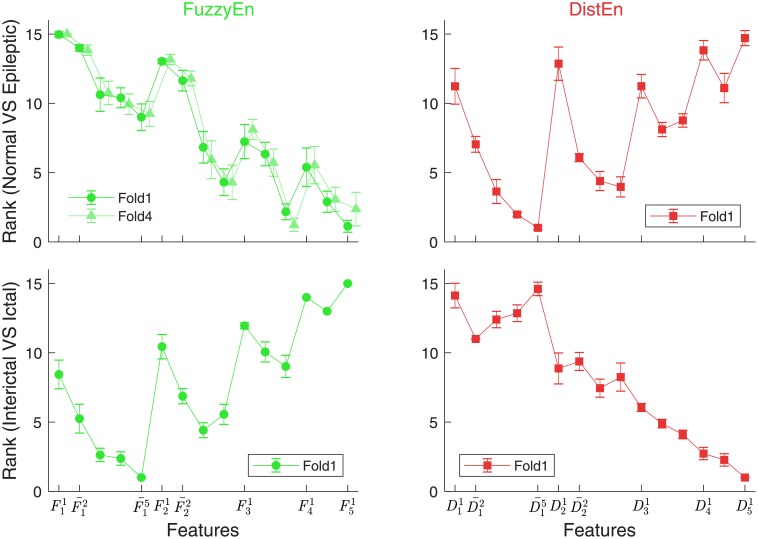
Rank matrix for different features. Subscripts in x-axes labels represent the length of windows and superscripts indicate how many windows were used or averaged if superscripts are larger than 1 (in this case a bar is also used to indicate the average). Because of limited space, x-axes are not fully labeled. For those without labels, the superscripts increase by 1 from left to right and are reset to 1 when subscripts change. Results are from fold 1 and are shown by mean (line) and standard deviation (error bar) across 250 ranks for each feature, except the upper left panel where results from fold 1 and fold 4 are shown.

The feature selection process has resulted in the single best feature from both FuzzyEn and DistEn features for both stages of classification. For the first stage classification (epileptic vs. normal), the lowest average rank value was obtained for FuzzyEn of one 5-s window ($F_{5}^{1}$; occurred in 3 runs) or averaged FuzzyEn of three 3-s windows ($\overset{-}{F_{3}^{3}}$; occurred in 2 runs), and averaged DistEn of five 1-s windows ($\overset{-}{D_{1}^{5}}$; occurred in all 5 runs), as shown in Fig 6. On the other hand, for the second stage classification (ictal vs. interictal), averaged FuzzyEn of five 1-s windows ($\overset{-}{F_{1}^{5}}$) and DistEn of one 5-s window ($D_{5}^{1}$) showed the lowest rank values for all 5 runs (Fig 6).

Table 1 shows the confusion matrix and the performance of the QD classifier in distinguishing the epileptic group from normal (task i, left part) and the ictal EEGs from interictal (task ii, right part). For task i (epileptic vs. normal), the FuzzyEn feature $F_{5}^{1}$ and the DistEn feature $\overset{-}{D_{1}^{5}}$ were used to train the first QD classifier and generate the cross-validation results. The results based on the combination of $\overset{-}{F_{3}^{3}}$ and $\overset{-}{D_{1}^{5}}$, or based on fold-specific best single FuzzyEn feature and $D_{1}^{5}$ (i.e., $F_{5}^{1}$ and $\overset{-}{D_{1}^{5}}$ for 3 folds while for the rest 2 folds, $\overset{-}{F_{3}^{3}}$ and $\overset{-}{D_{1}^{5}}$) were quite similar compared to those based on the selected combination (i.e., $F_{5}^{1}$ and $\overset{-}{D_{1}^{5}}$) and were reported in Supplemental Materials (S1 Table). On the other hand, for task ii (ictal vs. interictal), features $\overset{-}{F_{1}^{5}}$ and $D_{5}^{1}$ were used to train the second QD classifier and generate the cross-validation results. The overall accuracy we obtained for classifying the epileptic group from the normal group using both FuzzyEn and DistEn features ($F_{5}^{1}$ and $\overset{-}{D_{1}^{5}}$) is 92.80% (Table 1, left part) with sensitivity of 90.67% and specificity of 96.00%. On the other hand, classification of the ictal from interictal EEGs showed accuracy of 95.33% with sensitivity of 93.00% and specificity of 96.50% using FuzzyEn and DistEn features ($\overset{-}{F_{1}^{5}}$ and $D_{5}^{1}$). From the results, it is obvious that the best classification performance in both stages was achieved by combining the best performing features from DistEn and FuzzyEn. Individually, FuzzyEn showed better performance (accuracy = 90.40%) in classifying epileptic EEGs from normal than DistEn (accuracy = 84.00%). On the other hand, although both DistEn and FuzzyEn showed similar performance (accuracy of 77.33% and 78.67%) in classifying ictal from interictal EEG, DistEn showed more balanced results with higher sensitivity = 75.00%) than FuzzyEn (sensitivity = 66.00%).

**Table 1:** Confusion matrix and classification performance.

Classification task i				Classification task ii			
Confusion matrix (Features $F_{5}^{1}$ and $\overset{-}{D_{1}^{5}}$)				Confusion matrix (Features $\overset{-}{F_{1}^{5}}$ and $D_{5}^{1}$)			
	Epileptic	Normal	Actual		Ictal	Interictal	Actual
Epileptic	272	28	300	Ictal	93	7	100
Normal	8	192	200	Interictal	20	180	200
Predicted	280	220		Predicted	113	187	
Performance				Performance			
Features	Sensitivity	Specificity	Accuracy	Features	Sensitivity	Specificity	Accuracy
$F_{5}^{1}\;\overset{-}{D_{1}^{5}}$	90.67%	96.00%	92.80%	$\overset{-}{F_{1}^{5}}\;D_{5}^{1}$	93.00%	90.00%	91.00%
$F_{5}^{1}$	88.67%	93.00%	90.40%	$\overset{-}{F_{1}^{5}}$	66.00%	85.00%	78.67%
$\overset{-}{D_{1}^{5}}$	80.66%	89.00%	84.00%	$D_{5}^{1}$	75.00%	78.50%	77.33%

## Discussion

In this study, we proposed two protocols to analyze the entropy, i.e., FuzzyEn and DistEn, of EEGs with an aim of detecting epileptic activities based on nonlinear EEG dynamics. One protocol was to use a single analytical window with different window lengths. Another one was based on the average of multiple windows for each window length. Our motivation in proposing these two protocols was to attempt to find a definite “short” window length that could result in optimal classification performance to facilitate the online tracking of epileptic activities and even the prompt alarm of seizure onset and seizure attack in both ambulatory and in-hospital monitoring of EEGs. Our results suggest that:

1. Both FuzzyEn and DistEn could reach their optimal performance with window length *l* ≤ 5 *s*. Their performance was either maintained or even declined afterwards when the window length increased to above 5 s (Fig 4).
2. Averaging over approximately 5 or less windows for shorter window length (*l* ≤ 3 *s*) can improve the performance. For longer windows (e.g. *l* = 4 or 5 s), averaging either shows only slight improvement on the performance (Figs 5 and 6).
3. For better classification performance, features from both FuzzyEn and DistEn measures should be used to build the classifier. The best classification performance was achieved by using: i) FuzzyEn of one 5-s window and averaged DistEn of five 1-s windows for classifying normal from epileptic group; and ii) averaged FuzzyEn of five 1-s windows and DistEn of one 5-s window for classifying ictal from interictal EEGs.

Usually it is believed that longer data should be associated with better performance since: i) the longer data the higher the possibility of capturing the true dynamics; and ii) the statistical performance of algorithms is ordinarily better with a larger data set. Intriguingly, our results seem not to endorse this common expectation. We found that though increasing window length might help entropy measurements to achieve better performance, the effect was limited. Specifically, the best detection accuracy occurred with a certain window length, i.e., *l* = 5 *s* or less and the accuracy maintained or even declined afterwards with further increasing in window length. According to simulated bench-mark models, it is true that both FuzzyEn and DistEn show more consistent and robust performance with longer data [34, 43]. Thus the reason why longer (*l* > 5 *s*) EEGs were not accompanied by better FuzzyEn and DistEn performance may relate to the algorithm applied by the entropy measurements to reconstruct the dynamics, i.e., the delay embedding reconstruction. For synthetic data, it works fine because the dynamics simply follows the model. However, for real-world physiological data like EEGs, the dynamics is more complicated and changes frequently with time (time variant and non-stationary in nature) and this may challenge the homogenous nature of this algorithm.

In addition, it is also a widely accepted idea that averaging over multiple trials improves robustness. Our results were also only partially in support of this expectation. We found that averaging did help to improve the performance but it only affected the analysis based on shorter windows. For longer windows, the averaging effect either diminished or vanished. This phenomenon, to some extent underlines the window length effect, i.e., if the best performance has already occurred using a single window with a certain length, averaging based on multiple windows of the same length will not help improve the performance anymore. On the other hand, if the performance declined for windows with lengths larger than the “best-performance” window, averaging might help convert the declined accuracy and made it better for shorter windows (e.g. averaging over less than 5 windows).

When looking deep into the changes of FuzzyEn and DistEn values in different categories (see S1–S4 Figs), FuzzyEn values reduced whereas DistEn increased in the epileptic group as compared with the normal group, which is similar to what we reported in [6, 7]. Assumptions do exist that the brain may exhibit randomness in its normal state and change to deterministic chaotic dynamics during an ictal state. The higher FuzzyEn values in the normal group can be attributed to the stochastic dynamics of a normal EEG as FuzzyEn was developed to detect randomness or irregularity [43]. On the other hand, DistEn was found to increase in nonlinear deterministic dynamics [34]. The increase of DistEn values in the epileptic group can thus make sense because the dynamics of the EEG shifts to deterministic chaos in a seizure activity. Therefore, DistEn and FuzzyEn are likely to be sensitive to different EEG dynamics, offering a mechanistic answer for the question that combining FuzzyEn and DistEn improve each other’s performance. From purely the methodological viewpoint, FuzzyEn and DistEn differ from each other as FuzzyEn measures entropy rate—the increase of information with the increase of embedding dimension, whereas DistEn is Shannon entropy that measures the variety of patterns. The different characteristics FuzzyEn and DistEn catch also make it possible that they complement each other’s performance.

Since the reported classification performance here requires no more than 5-s EEG data, it is very promising for application in tracking epileptic activities and providing prompt feedback. So far as we know, it is the first study that has achieved such a high classification accuracy using purely short-term data, although there are a mass of publications that have reported almost ideal performance [27], they were indeed not based on short-term data. It should be noted that although Alam et al also reported a high accuracy based on features derived from ~1.5-s IMF of EEG data [8], their protocol, as we mentioned, was not truly short-term because the IMFs were obtained from long-term data. It is unclear and hard to predict whether the other way—construct IMFs based on ~1.5-s EEG and then derive the features—would be able to achieve similar performance or not.

In the database we used, each complete EEG recording is around 23.6 s. However, in our classifier, only the first 5-s segment from each was used. It is natural to ask whether the performance we reported is dependent on the segments or not. In other words, will the performance be different if other 5-s segments are used? One of our most recent studies [6] can potentially be used to answer this question. In that study, we applied three protocols to select different 5-s segments from the complete recording. The results indicated that the entropy measurements were segment-independent such that the performances of the three protocols were highly comparable to each other. Therefore, the effect of the selection of segment can be ruled out for the current study and it is more likely that the performance will remain similar if other 5-s segments are employed.

Study limitations. (1) It is worth noting that the database mixed scalp (applied for healthy subjects) and intracranial recordings (used for epileptic patients), which is therefore not perfect for testing different classification algorithms. It is possible that the amplitudes of intracranial recordings, overall, are higher partly due to the different locations of electrodes and the filtering mechanism of the skull. Before all formal data analyses, we have band-pass filtered all the raw EEGs (cut-off frequencies: 0.53–40 Hz) in order to minimize the possible filtering effect of the skull. Additionally, neither FuzzyEn nor DistEn is considered to be an amplitude-dependent measure since both measures perform amplitude normalization first. Therefore, the probability of similar vectors (for FuzzyEn) and the distribution of distances across all vectors (for DistEn) are performed in a comparable manner. (2) Given that the length of each EEG recording is fixed, fewer sliding windows are expected for larger window lengths. Thus, the observation that the averaging effect diminishes or vanishes for longer windows may come from the fact that fewer windows have been averaged across. However, since we only presented window lengths varying from 1s to 5s for the MP protocol (Fig 5), the potential effect of reducing sliding window numbers should be minimized, i.e., there are still 19 windows to be averaged for window length of 5s, which are highly comparable to the 23 windows for window length of 1s. However, it should be noted that this database, again, is not ideal for testing sliding window analysis for longer windows. In order for the proposed analysis framework to be further verified, studies based on long-term monitoring of EEG data are warranted.

## Supporting information

S1 Fig FuzzyEn of normal vs epileptic group (upper panel) and interictal vs ictal EEG (lower panel) calculated based on the single window protocol. Results are shown by median±interquartile range (IQR).
(EPS)

S2 Fig DistEn of normal vs epileptic group (upper panel) and interictal vs ictal EEG (lower panel) calculated based on the single window protocol. Results are shown by median±interquartile range (IQR).
(EPS)

S3 Fig FuzzyEn of normal vs epileptic group (left panels) and interictal vs ictal EEG (right panels) calculated based on the multiple windows protocol. Window lengths increase gradually from 1 s to 5 s from top to bottom. Results are shown by median±interquartile range (IQR).
(EPS)

S4 Fig DistEn of normal vs epileptic group (left panels) and interictal vs ictal EEG (right panels) calculated based on the multiple windows protocol. Window lengths increase gradually from 1 s to 5 s from top to bottom. Results are shown by median±interquartile range (IQR).
(EPS)

S1 Table Confusion matrix and classification performance. (PDF)
