# Supplementary material for: Detection of epileptic seizure based on entropy analysis of short-term EEG
Source: PLoS One. 2018 Mar 15;13(3):e0193691. doi: 10.1371/journal.pone.0193691 (PMC5854404; doi:10.1371/journal.pone.0193691)
Supplement: S1 Table — (PDF) [file pone.0193691.s005.pdf]

S1 Table. Confusion matrix and classification performance.

Classification task i			
Confusion matrix (F_5^1 and $\overline{D_1^5}$ in 3 folds and $\overline{F_3^3}$ and $\overline{D_1^5}$ in 2 folds)			
	Epileptic	Normal	Actual
Epileptic	273	27	300
Normal	8	192	200
Predicted	281	219	
Performance			
Features	Sensitivity	Specificity	Accuracy
F_5^1/ $\overline{F_3^3}$, $\overline{D_1^5}$	91.00%	96.00%	93.00%
Confusion matrix (in all 5 folds $\overline{F_3^3}$ and $\overline{D_1^5}$)			
	Epileptic	Normal	Actual
Epileptic	275	25	300
Normal	7	193	200
Predicted	282	218	
Performance			
Features	Sensitivity	Specificity	Accuracy
$\overline{F_3^3}$ $\overline{D_1^5}$	91.67%	96.50%	93.60%
